# Infantile transient smooth muscle contraction of the skin in a 1-month-old girl

**DOI:** 10.1016/j.jdcr.2023.09.035

**Published:** 2023-10-20

**Authors:** Emily L. Clarke, Noah Fanous, Nova Shu, Moise L. Levy

**Affiliations:** a Division of Dermatology, Department of Internal Medicine, Dell Medical School at The University of Texas at Austin, Austin, Texas; b The University of Texas Health Sciences Center at San Antonio, San Antonio, Texas; c Zitelli and Brodland P.C., Pittsburgh, Pennsylvania; d Department of Pediatrics, Dell Medical School at The University of Texas at Austin, Austin, Texas

**Keywords:** infantile transient smooth muscle contraction of the skin, pediatric dermatology

## Introduction

Infantile transient smooth muscle contraction of the skin is a rare occurrence involving episodic rippling and dimpling of the skin in infants. It is a benign phenomenon, thought to be caused by transient contraction of pilar smooth muscle due to infantile skin hypersensitivity or autonomic instability. Infantile transient smooth muscle contraction of the skin has no reported association with comorbid conditions or long-term sequelae, and over time, episodes seem to resolve entirely without affecting the child’s health or development. Herein, we present a case of infantile transient smooth muscle contraction of the skin in a healthy 1-month-old girl continuing through the age of 3 years old.

## Case presentation

**Fig 1:**
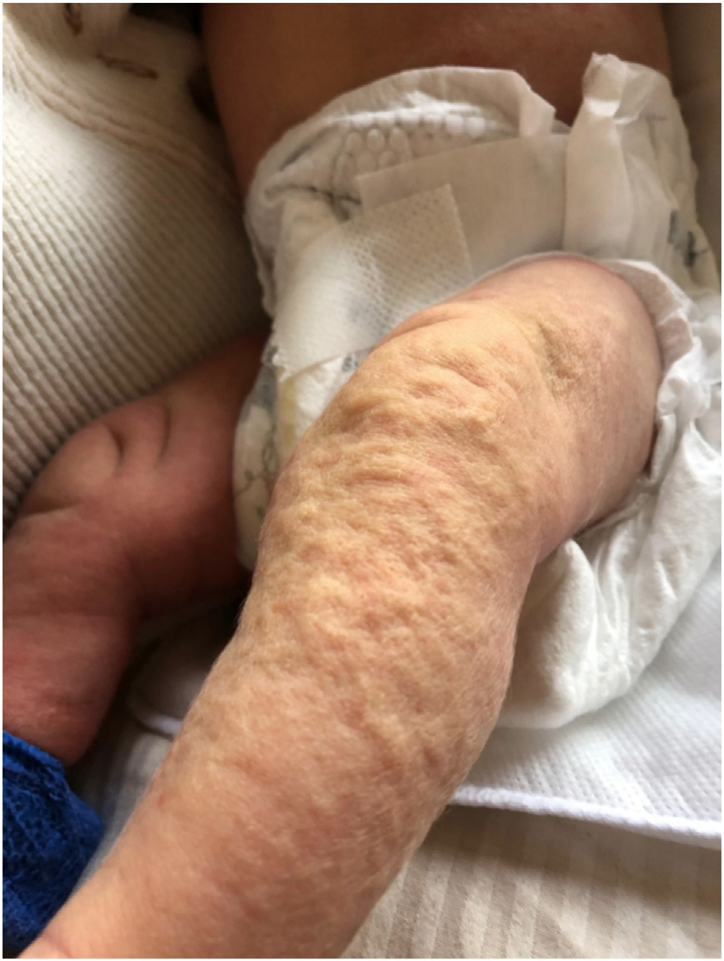
Rippling and blanching of the patient’s *left lower* extremity.

A 1-month-old healthy girl presented to the pediatric dermatology clinic with a history of episodic dimpling and rippling of the bilateral lower extremities, lasting for approximately 30 seconds and occurring several times each day. On occasion, the upper extremities were also involved. Her mother reported that these episodes began at birth and often coincided with shifting temperature, especially during diaper changes. In between these episodes, the infant’s skin appeared normal. On physical examination, the skin of the patient’s bilateral lower extremities appeared rippled and subtly blanched with no other pigmentary change or hypertrichosis (see Fig 1 and Supplementary Video 1, available via Mendeley at https://doi.org/10.17632/jy9nr475gb.1). The history and physical exam suggested a diagnosis of infantile transient smooth muscle contraction of the skin. Three years later, the patient’s episodes had significantly decreased in frequency over time but were still occurring occasionally on the lower extremities. At the time of follow up, the patient continued to grow and develop normally.

## Discussion

The term “infantile transient smooth muscle contraction of the skin” was first proposed to describe this episodic occurrence by Torrelo et al in 2013 after the authors encountered 9 newborns with transient rippling of the skin.^1^ This finding had previously only been reported in patients with suspected congenital smooth muscle hamartomas (CSMH), termed pseudo-Darier’s sign, which may be related to aberrant formation of the arrectores pilorum during fetal development.^2^, ^3^, ^4^ However, Torrelo et al proposed that infantile transient smooth muscle contraction of the skin is a distinct phenomenon from the findings associated with CSMH.^1^

Similar to our patient, many of the infants studied by Torrelo et al had symptoms bilaterally and no other evidence of CSMH such as pigmentary changes or hypertrichosis.^1^ Primarily healthy infants, both male and female, seem to develop this condition within the first few weeks of life and experience diminishing frequency of these episodes over time, contrary to a focal and persistent CSMH.^1^^,^^5^, ^6^, ^7^ Infantile transient smooth muscle contraction of the skin has been noted in several reports to favor the lower extremities, for reasons unknown.^1^^,^^7^ The episodes usually last seconds to minutes with normal-appearing skin between episodes.^1^ Consistent with the observation from our patient’s mother, triggers for the transient smooth muscle contraction include air, friction, bathing, and cold temperature.^1^^,^^5^ Infants with transient smooth muscle contraction of the skin appear to have no abnormalities of the skin or the dermal smooth muscle bundles on biopsy.^1^ One report noted moderate hyperplasia of dermal smooth muscle bundles on histopathologic exam.^5^ Although we did not pursue a biopsy for our patient, we suspect our patient would similarly have no abnormalities on skin biopsy. To date, there have been no long-term sequelae or negative health outcomes related to this condition reported, and the episodes of transient contractions resolve by the age of 2 in most children.^1^ Interestingly, although our patient’s episodes decreased in frequency over time, they continued through the age of 3 years old, which is rarely reported in the literature.

The mechanism for infantile transient smooth muscle contraction of the skin is largely unknown but may be related to autonomic instability or infantile skin hypersensitivity leading to transient contraction of the pilar smooth muscle.^1^ Newborns frequently experience other clinically benign, physiologic changes of the skin including acrocyanosis, cutis marmorata, and Harlequin color change.^8^ Infantile transient smooth muscle contraction of the skin may represent a similar benign physiologic skin finding.^1^ Previously there has also been debate in the literature about whether transient rippling of the skin is a form of myokymia, a neurologic term that describes undulating wave-like muscle movements or contractions.^9^^,^^10^ However, myokymia seems to primarily occur in adults with comorbidities rather than healthy infants and tends to involve deeper larger skeletal muscles rather than just the superficial smooth muscles within the skin.^9^^,^^10^

Infantile transient smooth muscle contraction of the skin is a rarely reported phenomenon in infants. It appears to be related to immaturity of the infant’s skin and autonomic nervous system and has no reported association with comorbid conditions or long-term sequelae. Over time, episodes are expected to resolve entirely without affecting the child’s long-term health or development. We present this case of infantile transient smooth muscle contraction of the skin to bring awareness to this rare entity so that physicians and parents can be reassured that no further workup or treatment is required if the patient is otherwise healthy.

## Conflicts of interest

None disclosed.
